# Durability of Basalt Fiber Fabric Under Simulated Lunar Extreme Thermal and UV Conditions: A Comparative Study with Natural Basalt Rock

**DOI:** 10.3390/polym18151876

**Published:** 2026-07-30

**Authors:** Jisiyuan Qin, Muhang Cai, Yutian Wang, Dan Sheng, Yunli Wang, Shan Jiang, Genyang Cao, Weilin Xu

**Affiliations:** State Key Laboratory of New Textile Materials and Advanced Processing, School of Textile Science and Engineering, Wuhan Textile University, Wuhan 430200, China; 15392869419@163.com (J.Q.); 2301230602@wtu.edu.cn (M.C.); 2301230503@wtu.edu.cn (Y.W.); dsheng@wtu.edu.cn (D.S.); ylwang@wtu.edu.cn (Y.W.); weilin_xu0@163.com (W.X.)

**Keywords:** basalt fiber fabric, polymer composite reinforcement, durability, lunar surface, extreme conditions

## Abstract

The extreme temperature cycling (−196 °C to 125 °C) and intense ultraviolet radiation on the lunar surface pose significant degradation risks to polymeric materials and their fibrous reinforcements. This work presents a comparative durability assessment between basalt fiber fabric and basalt rock powder after accelerated cyclic thermal shock treatments and UV exposure, aiming to establish a fundamental durability database for basalt fibers as candidate reinforcements for future polymer-matrix composites in lunar exploration. Multiscale characterizations reveal that regardless of the number of treatment cycles, the fiber surface morphology remains intact without cracking or etching. X-ray diffraction and Fourier-transform infrared spectroscopy confirm that the amorphous silicate network of the basalt fibers is well preserved, with no thermally induced recrystallization or compositional alteration. Thermogravimetric analysis further demonstrates that the treated fabrics retain the inherent thermal stability of pristine basalt, indirectly evidencing structural integrity. Importantly, the tensile strength of basalt yarns does not exhibit a cycle-dependent degradation trend, and the K/S colorimetric values remain stable even under combined thermal and UV exposure. Collectively, these findings confirm that basalt fiber fabric possesses exceptional resistance to lunar-mimetic aggressive environments, and its durability is comparable to that of natural basalt rock.

## 1. Introduction

Human beings have never stopped exploring outer space. The Moon, as the only extraterrestrial body humans have ever landed on, has drawn great attention. Due to the harsh conditions on the lunar surface, including extreme temperature variation, intense space radiation, and ultrahigh vacuum, stringent demands must be placed on the applied materials [1,2,3]. In terms of the flexible textiles used on the Moon, such as the space suits and the lunar module, the most commonly used high-performance fibers are polyimide [4], Kevlar [5], ultrahigh molecular weight polyethylene (UHMWPE) [6], carbon fibers [7], and their composites [8]. However, their manufacturing often involves large amounts of fossil fuel consumption and emission of carcinogenic substances, which has exerted serious pressure on both the environment and human health [9,10].

Basalt fiber is a type of high-performance inorganic fiber made from natural basalt, which is an important mafic volcanic rock originating from magmatic activity deep within the Earth [11,12]. Owing to its high specific strength, corrosion resistance, thermal stability, and compatibility with inorganic and polymeric matrices, basalt fiber has been used in the form of chopped fibers, continuous yarns, textile grids, reinforcing bars, and fabrics in polymer composites and civil-engineering structures. In particular, basalt textiles have attracted increasing attention in fabric-reinforced cementitious matrix (FRCM), textile-reinforced mortar (TRM), and other textile-reinforced inorganic-matrix systems for the external strengthening and seismic retrofit of masonry and concrete structures. Bencardino et al. [13] incorporated a coated basalt textile into a slag-rich hybrid alkali-activated mortar for masonry strengthening and compared its tensile and bond behavior with that of an equivalent lime-based system. Basalt fiber also offers potential environmental advantages over several conventional manufactured reinforcements. Because it is obtained directly from abundant volcanic rock through melting and fiber drawing, its production does not require the polymeric precursor synthesis associated with carbon and aramid fibers [14]. Moreover, a comparative life-cycle assessment of basalt- and glass-fiber-reinforced vinyl ester composites found that the basalt fiber composite exhibited a lower climate-change impact while maintaining competitive mechanical performance [15]. Basalt rock is also the main composition of the lunar Regolith [3,16]. Inspired by the in situ utilization principle, Xu’s group first prepared the national five-starred red flag of China using basalt fiber fabric as the base material, which has been successfully unfolded on the far side of the Moon on 3 June 2024 with the Chang’e-6 probe [17]. Through a unique spinning technique, this kind of ultrafine basalt fiber flag only weighs 11.3 g. Finally, it turned out that this flag exhibited excellent tolerance and stability on the Moon, thanks to the extraordinary chemical stability, good thermal resistance, and impressive mechanical properties of the basalt fibers.

Despite these sustainable and eco-friendly advantages, the long-term durability of basalt textiles under lunar conditions remains unclear. In the present study, basalt fiber fabric and basalt rock powder were subjected to the same extreme temperature cycling conditions. Because the melting and fiber-drawing processes substantially alter the crystalline structure of the original basalt rock [18,19,20,21,22], the fiber and powder were not regarded as structurally equivalent materials. Instead, two parallel within-material comparisons were conducted: the treated fiber samples were compared with the untreated fiber, while the treated powder samples were compared with the untreated powder. The cross-material comparison was therefore focused on the relative resistance of the two basalt-derived material forms to the applied treatment, rather than on their absolute structural or physical properties. Through multiple characterizations, including SEM, FTIR, XRD, TGA, tensile testing, and colorimetric analysis, we demonstrate that basalt fiber fabrics exhibit degradation behavior analogous to that of basalt rock, suggesting their capability to withstand extended service on the lunar surface. These findings not only confirm the intrinsic durability of basalt fibers but also provide a critical material database for the future design of basalt fiber-reinforced polymer composites intended for long-term extraterrestrial deployment, a topic of increasing relevance to the polymer composites community.

## 2. Experimental

### 2.1. Materials

Basalt fiber cloth with a plain weave structure and basalt powder with an average particle size of 30 mesh were purchased from Hubei Huierjie Basalt Fiber Co., Ltd. (Xiangyang, China) Liquid nitrogen was acquired from Wuhan Xiangyun Industry & Trade Co., Ltd. (Wuhan, China).

### 2.2. Extreme Condition Treatment

The present experimental protocol was designed as a controlled screening test focusing on two major lunar-relevant stressors: extreme temperature excursions and intense broadband irradiation. Therefore, the term “lunar-relevant conditions” is used in the present study to describe the selected laboratory conditions, and the results should be regarded as a preliminary durability assessment rather than a complete lunar-environment qualification.

(1) Extreme temperature (−196 °C~125 °C) treatment

**Figure 1:**
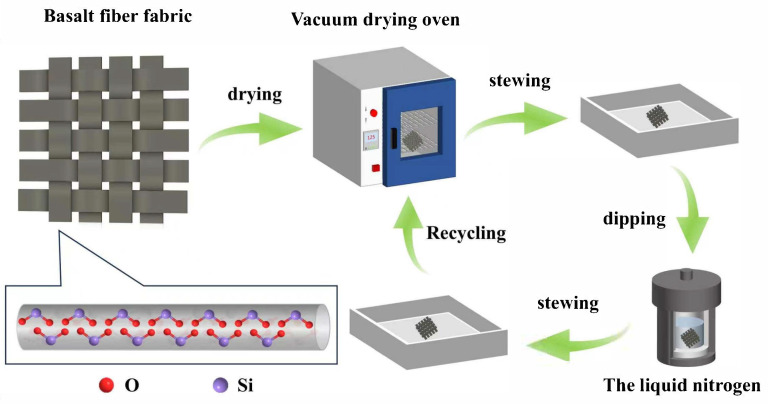
Flow chart of the extreme environment experiment.

To reproduce a representative lunar-relevant temperature range under controlled laboratory conditions, a thermal treatment covering a temperature range of −196 °C~125 °C was carried out on the basalt fiber fabrics and the basalt powder, as illustrated in Figure 1. For one specific cycle of the treatment, the basalt fiber fabric with a size of 20 cm × 4 cm or 10 g of the basalt powder was first placed in the vacuum drying oven (DZ-3BCIV, Tianjin Test Instrument Co., Ltd., Tianjin, China) at 125 °C for 24 h. After 30 min of standing in room temperature conditions, the sample was subsequently immersed in liquid nitrogen for another 24 h. For more cycles of this kind of high and low temperature treatment, the interval between each cycle was 30 min in room temperature conditions. In this work, a maximum of seven cycles were conducted, and the obtained samples were denoted as F-*x*c or P-*x*c (F refers to fabric, P refers to powder, c refers to cycle, and *x* refers to the number of extreme temperature treatments).

(2) Ultraviolet (UV) radiation treatment

To evaluate the color stability of the basalt samples under intense light exposure, the basalt fiber fabrics and basalt powder subjected to extreme temperature cycling were subsequently irradiated continuously for 24 h using a 125 W xenon lamp. The distance between the lamp outlet and the sample surface was maintained at 14.5 ± 0.5 cm. The incident irradiance at the sample position was measured using a CEL-NP2000 optical power meter (Beijing China Education Au-light Co., Ltd., Beijing, China) and was maintained at 150 mW cm^−2^, corresponding to 1500 W m^−2^. The resulting total incident radiant exposure was 129.6 MJ m^−2^.

No wavelength-selective UVA, UVB, or UVC band-pass filter was used; therefore, the treatment is described as broadband xenon-lamp irradiation rather than irradiation within a single UV region. The exposure was conducted in the air under ambient laboratory conditions without programmed humidity, water spray, or condensation. The 24 h period was selected for comparing the color stability of the different samples rather than as a direct simulation of a specific outdoor service duration.

### 2.3. Characterization Techniques

The surface morphology of the basalt fiber fabrics and basalt powder was observed using scanning electron microscopy (SEM, TESCAN MIRA LMS, Brno, Czech Republic). Prior to the test, all the samples were sprayed with gold under vacuum conditions, and the acceleration voltage was 10 kV.

Fourier transform infrared (FTIR) spectroscopy measurements were conducted using a spectrometer (VERTEX 70, Bruker Technologies, Berlin, Germany) equipped with an attenuated total reflection accessory. The scanning range for the measurements was set from 4000 to 400 cm^−1^, with a resolution of 8 cm^−1^.

The crystal structure of the basalt fiber fabric and the basalt powder was analyzed using an X-ray diffractometer (XRD, EMPYREAN, Panalytical, Almelo, the Netherlands). The X-ray utilized was a Cu-Kα ray with a wavelength of 0.154 nm, and the scanning speed was set at 5°/min. The scanning angle ranged from 5° to 75°. The apparent crystallinity index was calculated from the XRD patterns using Jade software (Jade 6.5) after background subtraction and peak separation. The crystalline and amorphous contributions were determined by integrating the areas of the resolved crystalline peaks and the broad amorphous scattering region, respectively. The apparent crystallinity index, Xc, was calculated according to Equation (1):

(1) $$Xc(\%)=\frac{Ac}{Ac+Aa}*100$$

where Ac is the integrated area of the crystalline diffraction peaks, and Aa is the integrated area of the amorphous scattering region.

The thermal stability of the basalt fiber fabric and the basalt powder was evaluated using a thermogravimetric analyzer (Thermo Fisher Scientific, Waltham, MA, USA) under a nitrogen atmosphere. The heating rate was set at 5 °C/min.

The color characteristics of the samples, mainly the K/S values, were measured using a computer color matching system (Color i7, X-Rite, Grand Rapids, MI, USA) with a 10° standard observer and D65 illuminant. Each sample was tested five times, and the results were averaged. Relative color strengths (K/S values) were measured on the basis of the Kubelka–Munk equation:

(2) $$K/S=\frac{{\left(1-R\right)}^{2}}{2R}$$

where K is the coefficient of absorption, S is the scattering coefficient, and R is the reflectance of the dyed filament.

The tensile properties of the basalt fiber yarns unraveled from the fabrics were measured using an Instron 5943 universal testing machine (Instron, Norwood, MA, USA) with reference to ASTM D2256/D2256M-21 [23], with modifications to the gauge length and crosshead speed. A gauge length of 50 mm and a crosshead speed of 20 mm/min were used. Ten specimens were tested for each sample, and the breaking tenacity, elongation at break, and initial modulus are reported as the mean ± standard deviation.

## 3. Results and Discussions

### 3.1. Surface Morphology

**Figure 2:**
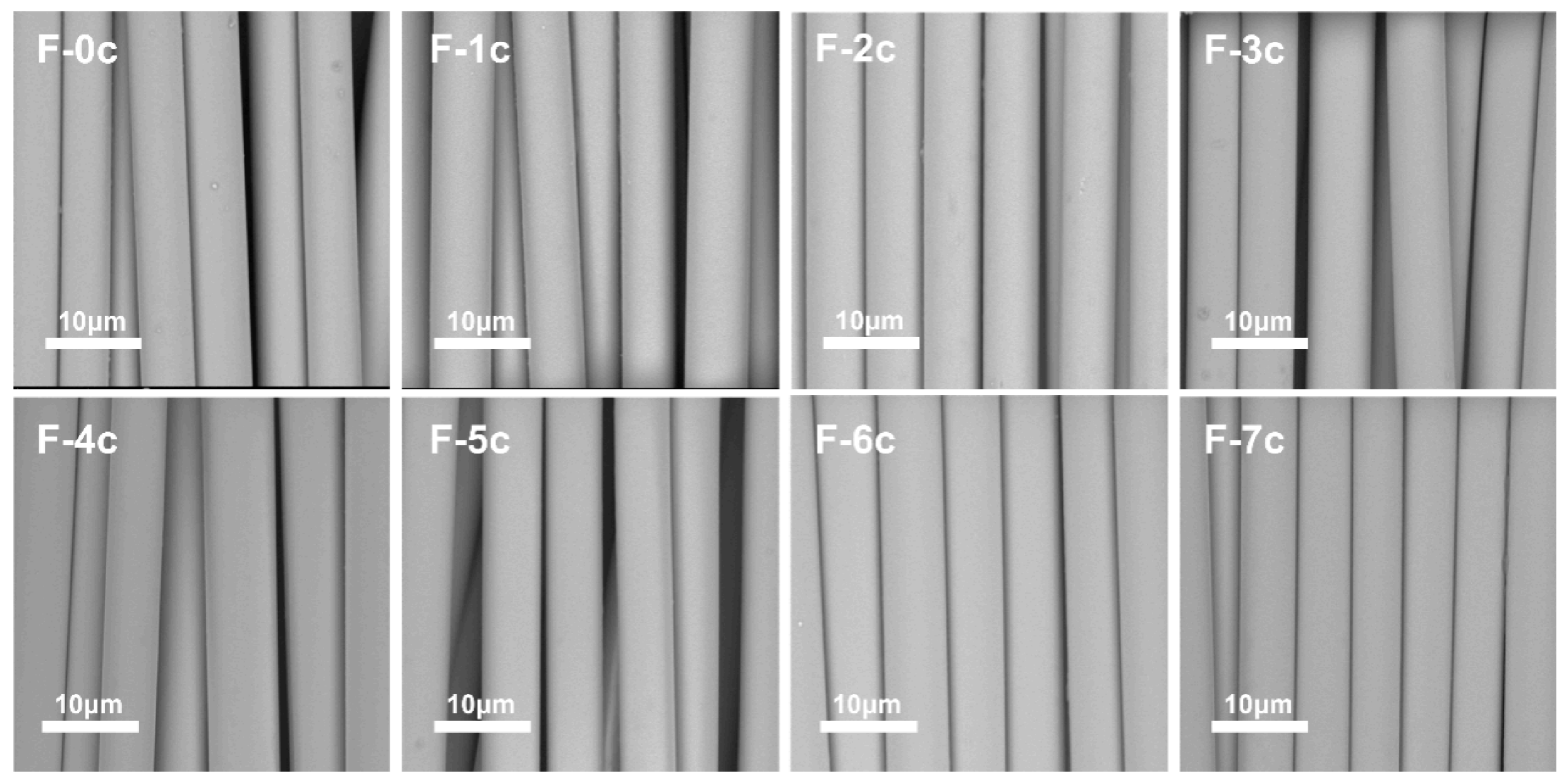
SEM images of the basalt fiber fabrics with different cycles of extreme temperature treatment.

**Figure 3:**
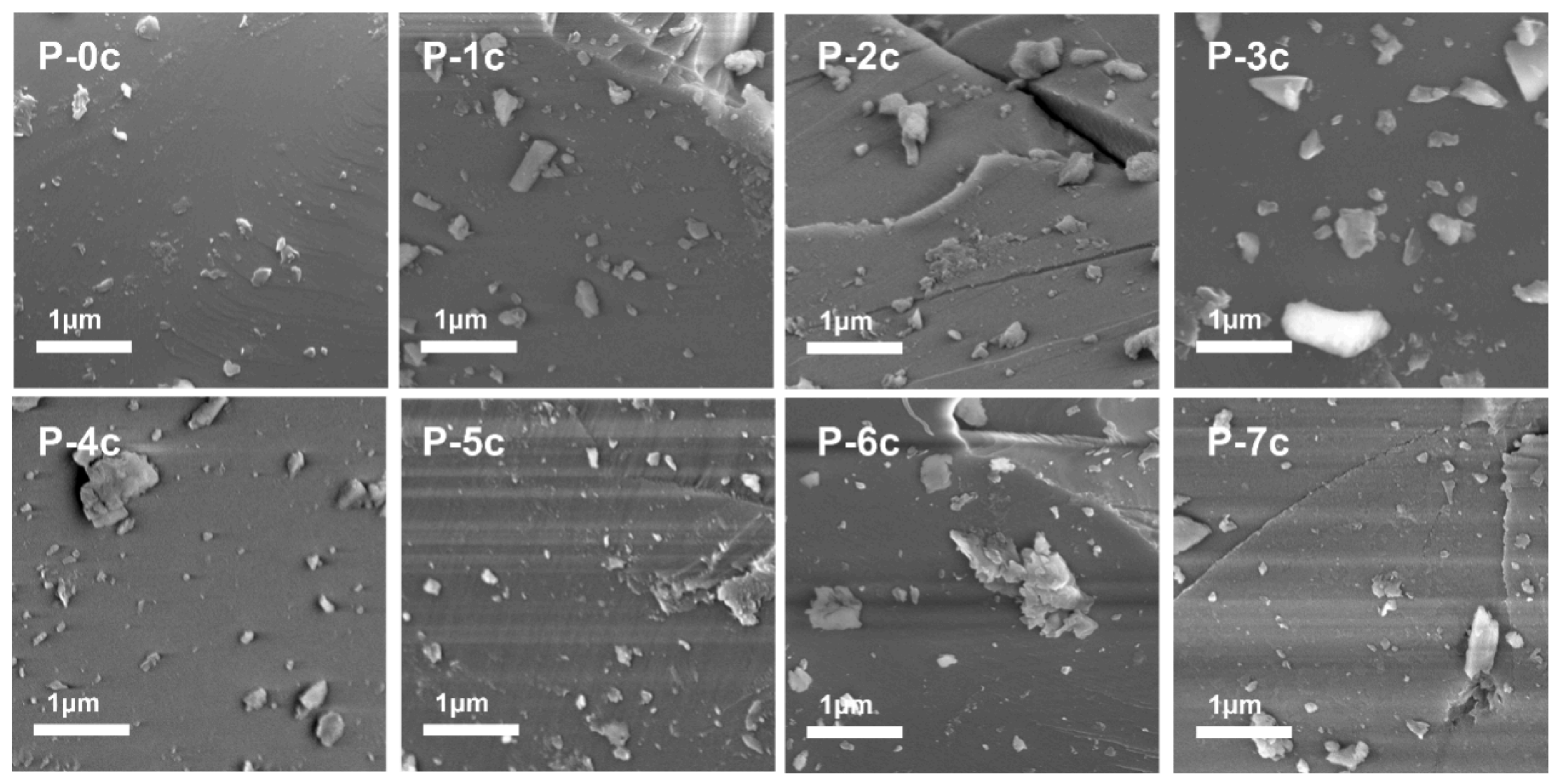
SEM surface images of the basalt powder with different cycles of extreme temperature treatment.

Starting from the comparison of the surface morphology, Figure 2 and Figure 3 respectively show the SEM images of the basalt fiber fabric and basalt powder before and after extreme temperature treatment. It can be seen from Figure 2 that the untreated fabric displays a consistent and well-aligned network of basalt fibers, and individual fibers exhibit a smooth and uniform surface [24]. The SEM examination was primarily intended to identify visible physical damage caused by repeated thermal contraction and expansion, including surface cracking, pitting, etching, local spalling, filament breakage, and diameter discontinuity. The untreated fibers exhibit smooth and continuous surfaces. After the high and low temperature (−196 °C~125 °C) treatment, no obvious changes can be observed from the appearance regardless of the number of cycles, except for the inherent variation in thickness between fibers, indicating that the extreme temperature conditions have very limited effect on the surface morphology of the basalt fibers.

**Figure A1:**
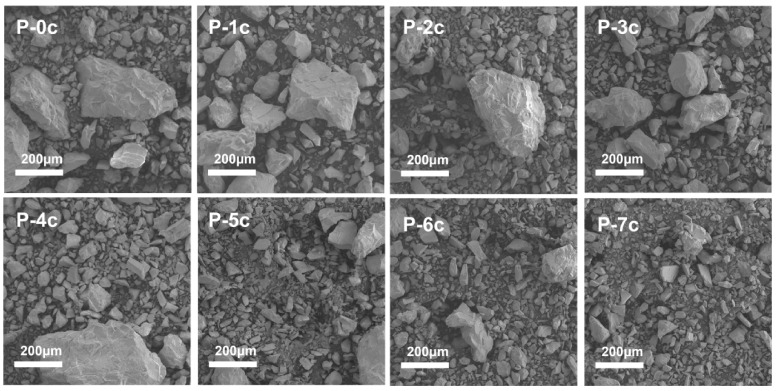
SEM surface images of the basalt powder with different cycles of extreme temperature treatment under low magnification.

In the meantime, the SEM images have also been taken for basalt powder, as shown in Figure 3. As expected, the low-magnification images show a relatively chaotic scene (Figure A1). Basalt particles with varying sizes are randomly distributed. Focusing on the fairly flat position under higher magnification in Figure 3, except for some tiny particles distributed on the top, a rather smooth rock surface can be revealed. Although some stripes on the surface can be seen for P-2c, P-5c, and P-7c, this phenomenon can be attributed to the structural features of the basalt itself, rather than the extreme temperature treatment. Other than these kinds of surface differences between the samples treated with different cycles, no significant etching or roughness changes are confirmed. Therefore, it can be concluded that the influence of extreme temperatures on the surface morphology of the basalt powder is also negligible.

### 3.2. Crystalline Properties

**Figure 4:**
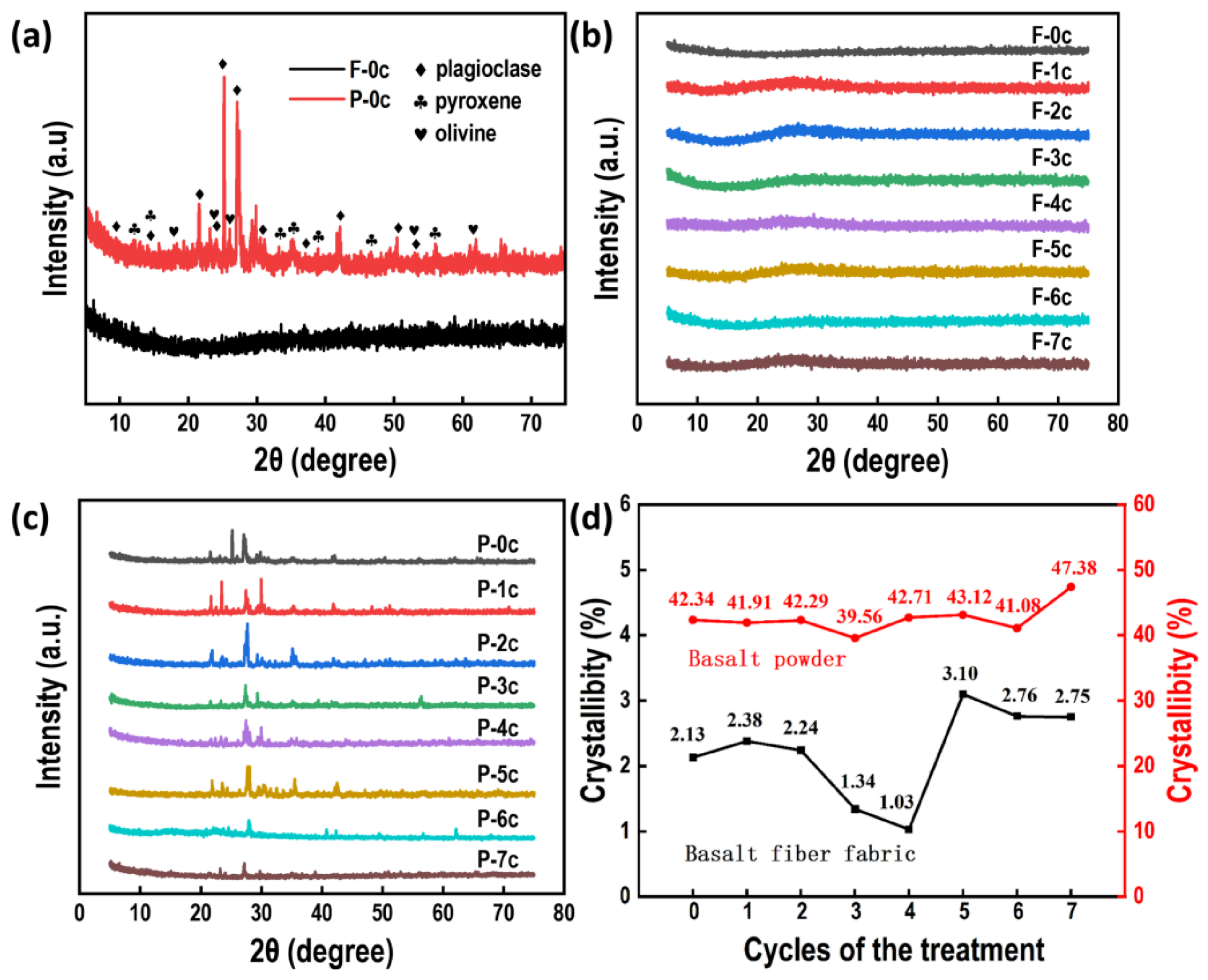
(**a**) XRD patterns for the untreated basalt samples; (**b**) XRD patterns for the basalt fiber fabrics with different cycles of extreme temperature treatment; (**c**) XRD patterns for the basalt powder with different cycles of extreme temperature treatment; (**d**) apparent crystallinity indices calculated by XRD peak-area separation using Jade software.

XRD measurements were conducted to analyze the crystalline structures of all the basalt samples before and after the extreme temperature treatment, as presented in Figure 4. Figure 4a exhibits the XRD patterns of the untreated basalt fiber fabrics and the basalt powder. A great difference can be observed between the basalt fibers and the basalt powder. Compared with a broad, diffuse scattering profile of the basalt fiber sample, more complex and sharper peaks can be found for the basalt powder, indicating much higher crystallinity of the basalt powder. After careful analysis, the mineral composition of the basalt powder mainly belongs to plagioclase, pyroxene, and olivine [25,26]. Considering the basalt fiber is obtained by heating basalt rock to a temperature between 1450 °C and 1500 °C, followed by the drawing process, the large long-range crystalline structure of the basalt rock is destroyed, leaving behind amorphous phases. The apparent crystallinity indices calculated from the XRD patterns are summarized in Figure 4d. The untreated basalt powder (P-0c) exhibited an apparent crystallinity index of 42.3%, whereas the untreated basalt fiber fabric (F-0c) showed a substantially lower value of 2.1%. This pronounced difference is attributed to the melting and rapid fiber-drawing processes used in basalt fiber production, which disrupt the long-range ordered mineral structures of the original basalt rock and generate a predominantly amorphous silicate network. After cyclic extreme temperature treatment, the individual apparent crystallinity indices are summarized in Figure 4d. Although minor fluctuations were observed, neither sample group exhibited a monotonic increase or decrease with increasing treatment cycles. Therefore, the observed variations are more reasonably attributed to sample heterogeneity and uncertainties associated with peak separation than to thermally induced crystallization or amorphization. In contrast, the XRD peak intensities for different basalt powder samples vary, although the peak positions remain almost constant, which can be attributed to the heterogeneity of the basalt ores.

However, the crystallinity for different basalt powder samples still has a slight fluctuation. From all these results, no significant causal relationship was found between the change in the crystallinity and the cycles of the applied treatment. At the atomic scale, basalt fiber is composed mainly of a disordered aluminosilicate network constructed from interconnected SiO_4_ tetrahedral units [27]. The maximum treatment temperature of 125 °C is insufficient to provide the atomic mobility required for extensive rupture and reconstruction of the strong Si–O network bonds, long-range diffusion, nucleation, and crystal growth, while exposure to −196 °C further suppresses atomic motion. Repeated thermal expansion and contraction may reversibly alter local bond angles, inter-tetrahedral distances, free volume, and residual stress; however, these short-range structural adjustments are insufficient to generate permanent long-range ordering. The small apparent variations have different experimental origins for the two sample forms. For the predominantly amorphous fibers, the very low crystallinity index is sensitive to background subtraction and separation of weak crystalline contributions from the broad amorphous halo, as well as to fabric placement and the irradiated sample volume. For the basalt powder, natural mineralogical heterogeneity, particle-size distribution, packing density, and preferred orientation can change relative peak intensities without changing the crystalline phases. Consequently, the observed fluctuations are attributed to sample heterogeneity and normal analytical uncertainty rather than to a genuine cycle-dependent structural transformation.

### 3.3. Surface Chemical Composition

**Figure 5:**
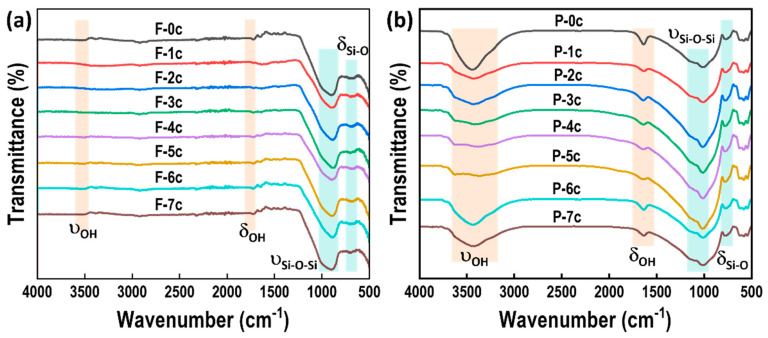
FTIR spectra for the (**a**) basalt fiber fabrics and (**b**) basalt powder with different cycles of extreme temperature treatment.

FTIR spectroscopy was employed to elucidate the surface chemical composition induced on the basalt fiber fabric and basalt powder before and after extreme temperature treatment, as shown in Figure 5. Focusing on the FTIR curves of the original basalt fiber fabric and basalt powder, the former exhibits a prominent absorption band centered at approximately 1000 cm^−1^, which can be attributed to the Si-O-Si stretching vibrations, while a less intense peak near 700 cm^−1^ can be assigned to Si-O bending modes. These key characteristic absorption bands originate from the inherent silicate networks in the basalt fibers. In addition, a broad weak absorption band at approximately 3400–3500 cm^−1^ and a weaker band centered at approximately 1640 cm^−1^ are observed. The broad high-wavenumber band is assigned mainly to the O–H stretching vibrations of hydrogen-bonded adsorbed water, with a possible overlapping contribution from surface silanol groups (Si–OH). In contrast, the band at approximately 1640 cm^−1^ is specifically attributed to the H–O–H bending vibration of molecularly adsorbed water. Water-bending vibrations at approximately 1620–1650 cm^−1^ have been widely reported for water adsorbed on silica-based surfaces, and a band near 1640 cm^−1^ has also been identified as the H–O–H bending mode in basaltic materials [28,29,30,31]. By contrast, the original basalt powder has similar, yet more distinct peak features, especially for the adsorption band of the O-H bond. Moreover, a slight shift towards the higher wavenumbers of the Si-O and O-Si-O vibrations for the original basalt powder can also be found [32]. This shift is primarily attributed to differences in mineralogical and local structural states. The powder retains crystalline plagioclase, pyroxene, and olivine phases with relatively well-defined Si-O bonding environments, whereas melting and rapid drawing produce a predominantly amorphous fiber structure with a broader distribution of Si-O bond angles [33,34]. In silicate systems, more polymerized, bridging-oxygen-rich structural units generally contribute at higher wavenumbers, and the principal Si–O band of basaltic glasses has also been reported to shift toward higher wavenumbers with increasing SiO_2_ content [35]. Thus, the observed shift likely reflects the combined effects of preserved crystalline phases, local network connectivity, and possible compositional differences. Particle-size and ATR-contact effects may provide a minor additional contribution [36].

When comparing different fabric samples after extreme temperature treatment, both the main peak position and peak intensity showed minimal changes, while the adsorption bands of the O-H vibrations showed a noticeable decrease for the treated basalt powder samples, indicating that adsorbed water on the surface evaporates during extreme temperature treatment.

### 3.4. Thermal Properties

**Figure 6:**
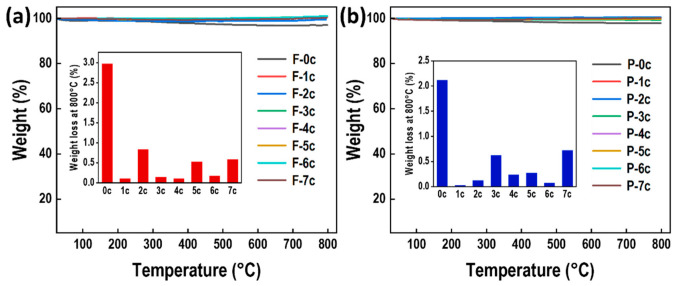
TGA curves for the (**a**) basalt fiber fabrics and (**b**) basalt powder with different cycles of extreme temperature treatment (insert: the weight loss at 800 °C).

To elucidate the effect of extreme temperature conditions on the thermal stability and decomposition behavior of the basalt samples, TGA tests were performed, and the curves are depicted in Figure 6. Figure 6a and Figure 6b respectively show the TGA curves of the basalt fiber fabrics and the basalt powder, and the weight loss at 800 °C has been particularly highlighted. Impressively, over the entire temperature range of 0–800 °C, no significant change in the sample weight can be observed for both figures, indicating the superb thermal stability of the basalt samples [37]. However, it should be noted that the weight loss for both original basalt samples exceed the treated samples after extreme temperature conditions. Taking the final weight loss at 800 °C as an example, the value for the original basalt fiber fabric is ~3%, while the other treated samples only exhibit values of <1%. A similar case also applies to basalt powder samples. This difference is mainly attributed to moisture removal during the preceding extreme temperature treatment. Because each cycle included vacuum heating at 125 °C for 24 h, a substantial fraction of the physically adsorbed water and other weakly bound water-related species had already been removed before TGA. This interpretation is consistent with the decreased O–H stretching and H–O–H bending bands observed by FTIR after treatment. Therefore, the lower mass loss of the treated samples reflects their reduced moisture content.

### 3.5. Mechanical Performance

**Figure 7:**
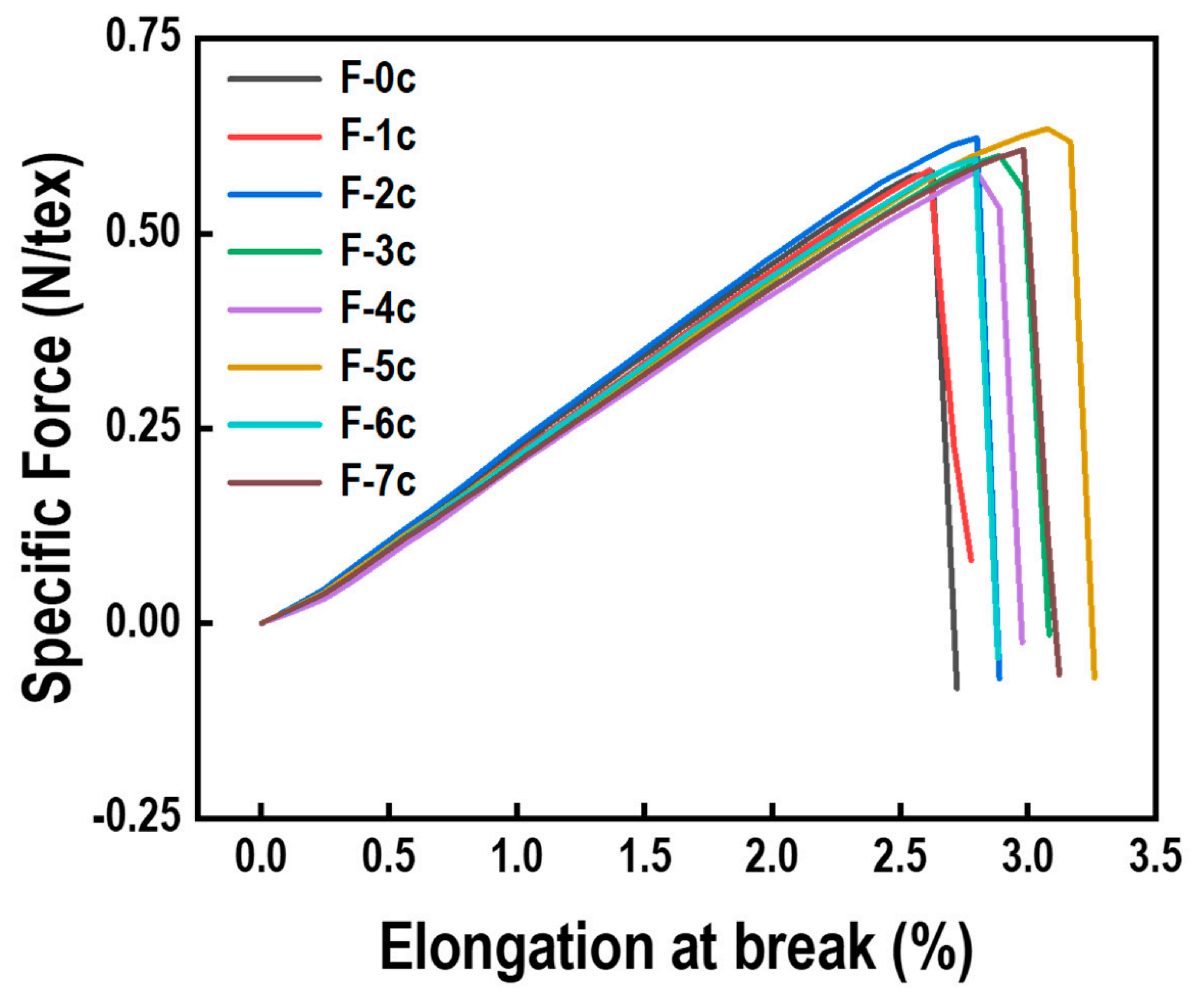
The tensile strength of the basalt fiber yarns with different cycles of extreme temperature treatment.

The mechanical performance is also an important indicator to reflect the durability under extreme conditions. However, direct tensile testing of woven fabrics is affected not only by the intrinsic properties of the basalt yarns but also by fabric-specific factors, including yarn crimp, interlacing geometry, yarn–yarn friction, local areal-density variation, yarn slippage, specimen alignment, and gripping conditions. These factors can increase the variability of the measured fabric response and may obscure relatively small effects associated with extreme temperature cycling. Therefore, the tensile strength of the basalt fiber yarns unraveled from the fabrics after extreme temperature treatment was analyzed instead, and the results are depicted in Figure 7. Considering the inherent unevenness of the yarn, ten measurements were taken for each sample and averaged. Except for the consistent initial modulus, some differences can be found between the basalt fiber yarns with different treated cycles, including breaking elongation and breaking stress. However, there is no positive correlation between the tensile performance and the treated cycles.

Table 1 summarizes the detailed mechanical properties of basalt fiber yarns subjected to different cycles of extreme temperature treatment, including breaking strength, elongation at break, and initial modulus, along with their corresponding coefficients of variation (CV). Overall, the CV values for all samples remain at a relatively low level, indicating good repeatability and statistical reliability of the test data, which can faithfully reflect the mechanical performance of the samples. However, with increasing treatment cycles, none of the three parameters—breaking strength, elongation at break, or initial modulus—exhibits a monotonic increasing or decreasing trend. For instance, although the F-5c sample shows slightly higher values in breaking strength (63.65 cN/tex) and elongation at break (3.26%) compared to others, no systematic differences are observed between adjacent cycle numbers, and the fluctuations fall within the normal range attributable to the inherent unevenness of the yarns themselves. The initial modulus does not exhibit a consistent monotonic dependence on the number of treatment cycles. A progressive decrease in modulus would require the accumulation of permanent defects, such as microcracks or disruption of the silicate network, whereas a progressive increase would require a continuous stiffening mechanism, such as crystallization or structural densification. Neither mechanism is supported by the SEM, XRD, or FTIR results. The observed fluctuations are more reasonably attributed to specimen-to-specimen variations in filament alignment, yarn waviness, initial slack, gripping conditions, the number of load-bearing filaments, and local defect distribution, to which the initial slope of the stress–strain curve is particularly sensitive. These findings suggest that the extreme temperature cycling treatment is not the dominant factor affecting the mechanical properties of basalt fiber yarns; rather, the intrinsic structural heterogeneity of the yarns plays a more critical role in determining the test results. Therefore, it can be concluded that the extreme temperature conditions do not cause cumulative damage to the mechanical performance of basalt fibers.

### 3.6. Color Degradation

**Figure 8:**
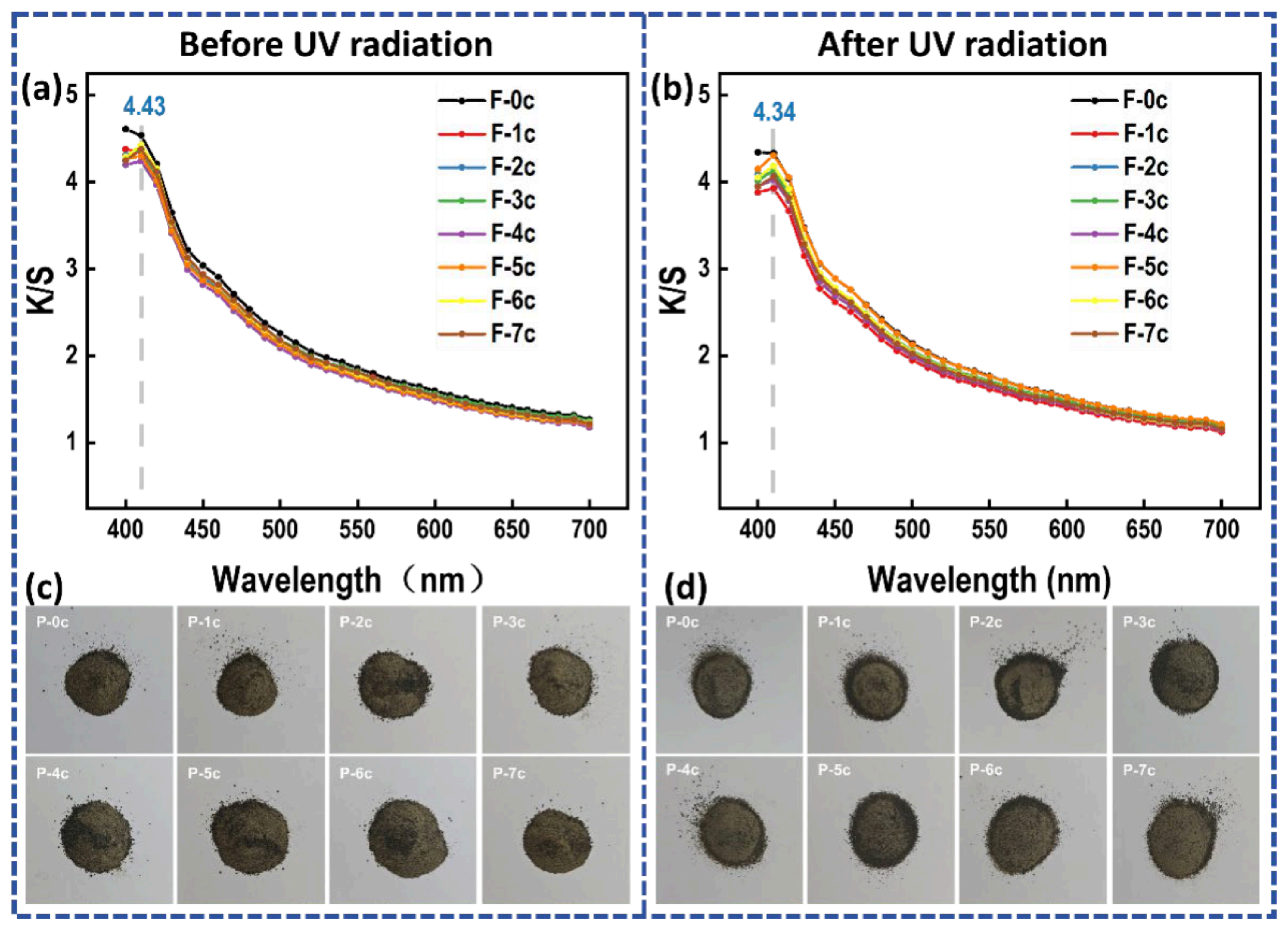
Color property for the treated basalt samples before and after UV radiation: (**a**) K/S values for the treated basalt fabrics before UV radiation, (**b**) K/S values for the treated basalt fabrics before UV radiation, (**c**) intuitive photos for the basalt powder before UV radiation, (**d**) intuitive photos for the basalt powder after UV radiation.

Color stability was evaluated as an auxiliary indicator of irradiation-induced surface alteration rather than as independent evidence of overall material durability. Color changes are generally sensitive to photochemical degradation in conventional textiles because many organic dyes contain chromophoric structures that can be disrupted by ultraviolet radiation. In view of the great impact of the radiation on the color property, an ultraviolet radiation procedure with a duration of 24 h was further carried out on the basalt fibers and the basalt powder, followed by the extreme temperature treatment. After that, the K/S values of the basalt fiber fabrics before and after UV radiation were recorded to investigate the color degradation, as shown in Figure 8a,b. Due to the intrinsic brown color of basalt ores, there is a continuous decreasing curve for the K/S value, with no obvious peaks. However, the degradation trend for the color depth can still be found. This limited color variation is consistent with the optical characteristics of basalt fibers reported in the literature. Ruffen and Mahltig [38] demonstrated that basalt fibers strongly reduce ultraviolet transmission, particularly below 300 nm, owing to the UV absorption of their inorganic constituents. Barczewski et al. [39] further reported only small color variations after accelerated UV aging for polyurea coatings containing basalt microfibers or basalt powder, with maximum ΔE variations of approximately 1.98 and 0.96, respectively. In addition, basalt fiber-reinforced laminates have been reported to exhibit less pronounced UV-induced degradation than comparable glass-fiber-rich systems [40]. Similar to the above TG curves, the K/S curves for all the treated samples almost overlap with the original ones, and little difference can be found between Figure 8a,b. This indirectly demonstrates that the surface color of basalt fiber fabrics exhibits exceptional resistance to ultraviolet radiation and extreme temperature conditions.

Because it is difficult to reflect the color property of the basalt powder with the computer color matching system, intuitive photos were taken for the powder samples before and after UV radiation, as shown in Figure 8c,d. Similar to the fabric samples, no obvious color degradation was observed, indicating that the surface color of the basalt ores apparently did not change under the extreme conditions.

## 4. Conclusions

This work systematically evaluated the durability of basalt fiber fabric under simulated lunar extreme conditions (−196 °C to 125 °C thermal cycling combined with UV radiation) through a comparative study with natural basalt rock powder. The key findings are summarized as follows:

(1) Both basalt fiber fabric and basalt powder exhibit exceptional resistance to cyclic thermal shock, with no detectable surface degradation, compositional alteration, or crystalline transformation across multiple characterization techniques (SEM, XRD, FTIR, and TGA).
(2) The tensile strength of basalt fiber yarns shows no cycle-dependent degradation, and the surface color remains stable under combined thermal and UV exposure, confirming the structural and esthetic integrity of the fabric under simulated service conditions.
(3) Critically, the degradation behavior of basalt fiber fabric is analogous to that of basalt rock across all evaluated metrics, supporting the conclusion that the fiber’s durability is comparable to that of its natural mineral precursor.

Although the seven-cycle protocol imposed repeated thermal excursions, it does not provide a quantitative prediction of long-term service life. The absence of a progressive degradation trend suggests that a modest extension beyond seven cycles under the same isolated laboratory conditions would be unlikely to cause an abrupt performance change. Nevertheless, longer-duration testing and coupled thermal–vacuum–radiation exposure are required before long-term lunar serviceability can be established.

Despite this limitation, the present results provide an experimental basis for extending the in situ resource utilization potential of lunar basalt beyond conventional regolith-based construction materials. In particular, lunar basalt may potentially be processed into fibers and further fabricated into textiles and fiber-reinforced composite materials directly on the lunar surface. Such a material pathway could reduce the demand for transporting reinforcement materials from Earth and may contribute to the sustainable development of future extraterrestrial infrastructure and long-duration space missions.

## Figures and Tables

**Table 1:** Mechanical property table of the sample.

Sample	Breaking Strength (cN/tex)	Elongation at Break (%)	Initial Modulus (cN/dtex)	CV
F-0c	58.09 ± 0.77	2.72 ± 0.03	235.04 ± 2.35	1.00%
F-1c	58.45 ± 0.25	2.78 ± 0.19	235.64 ± 10.75	4.56%
F-2c	62.56 ± 3.19	2.89 ± 0.13	247.12 ± 2.14	0.87%
F-3c	60.27 ± 2.56	3.09 ± 0.17	227.86 ± 4.93	2.16%
F-4c	57.97 ± 1.68	2.98 ± 0.02	226.97 ± 6.40	2.82%
F-5c	63.65 ± 2.44	3.26 ± 0.15	220.94± 2.83	1.28%
F-6c	59.78 ± 1.62	2.89 ± 0.12	233.55 ± 2.74	1.17%
F-7c	60.99 ± 3.36	3.12 ± 0.13	229.76 ± 10.77	4.69%

## Data Availability

The original contributions presented in this study are included in the article. Further inquiries can be directed to the corresponding author(s).
